# Ectopic Supernumerary Tooth in Nasal Septum: A Case Study

**Published:** 2013-06

**Authors:** Saleh Mohebbi, Oveis Salehi, Sedighe Ebrahimpoor

**Affiliations:** 1 *Department of Otorhinolaryngology.Tehran University of Medical Sciences,Tehran,Iran. *; 2 *Department of Otorhinolaryngology.Hormozgan University of Medical Sciences,Bandarabbas,Iran.*; 3 *Cell and molecular biology,biochemistMsc,Research center of otolaryngology –head and neck surgery,Iran university of medical sciences,Tehran,Iran.*

**Keywords:** Ectopic tooth eruption, Septal deviation, Supernumerary, Tooth

## Abstract

**Introduction:**

Nasal teeth eruption is a rare phenomenon. The variability of symptoms and generic history makes the diagnosis difficult. This difficulty is more challenging when the tooth is placed in the depth of septum.

**Case Report:**

Our case is an example of this problem. Herein, we present a case of intraseptal tooth with nasal obstruction and septal deviation and recurrent sinusitis. We present preoperative imaging.

**Conclusion:**

Great suspicion may helpful for preoperative diagnosis and good deciding.

## Introduction

Ectopic teeth can be seen in different areas of the body. Nasal teeth eruption is a rare phenomenon (1). It can be accompanied by variable general symptoms such as external body sensation, nasal obstruction, purulent malodor nasal discharge, epistaxis, epiphora, headache, rhinolithiasis, external nasal deformity and nasolacrimal duct obstruction (1-6). The variability of symptoms and generic history makes the diagnosis difficult. This difficulty may lead to delayed detection and treatment, therefore complications may occur. This difficulty is more challenging when the tooth placed in the depth of septum. According to this problem, we present a case of intraseptal tooth with nasal obstruction and septal deviation and recurrent sinusitis.

## Case Report

A 19 years old male patient, showed up to our clinic with bilateral nasal obstruction, open mouth breathing and intermittent headache, which was more dominant in the mornings. He also complained about episodes of recurrent sinusitis in the past. But he did not complain about nasal discharge at the moment.

Neurological exams were normal. No tenderness detected at the time of compression of cheeks. His throat was mildly erythematous; no significant postnasal discharge (PND) was detectable at the time of examination. Rhinologic exam revealed a septal deviation, which was spurred like.

**Fig1:**
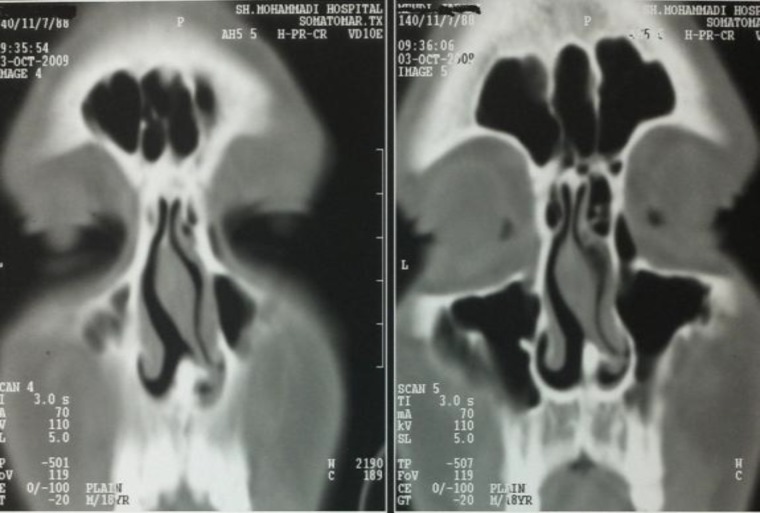
Preoperative CT scan of paranasal sinus.show a bright object in floor of nasal cavity in septum,also severe septal deviation

CT scan showed severe septal deflection and deviation, and a density in the floor of nasal septum that was noted in the second look (Fig. 1).

**Fig 2:**
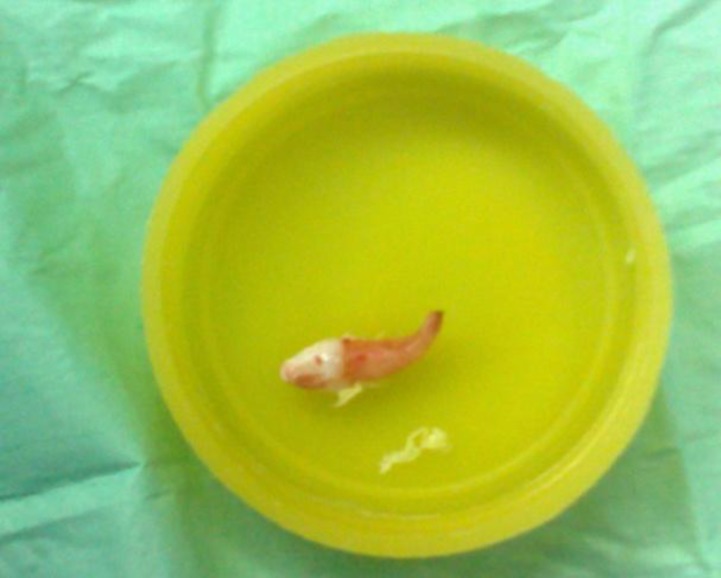
Extracted tooth after surgery,resemble a canin teeth

According to the severe septal deviation we decided to perform conventional septoplasty. Killian incision performed and submucoperi- chondrial flap was held, we saw a whitish hard object while dissecting quadriangular cartilage. We pulled it out after septal floor osteotomy; it was relatively developed tooth, which was highly resembled to a canine tooth. It had one root which was not too firm (Fig. 2). Not any missed tooth was noted in oral exam, and dental count was normal. After removing the tooth, septoplasty completed. The post operative course was passed without any complication or morbidity. The patient was free of symptoms 10 days after the surgery.

## Discussion

Eruption of ectopic teeth can be seen in about 0.5% of population (1,7). The most common site for this phenomenon is upper incisor area which is called mesiodens (1,7). Intranasal tooth is a rare condition (7). This condition has been reported by smith et al., for the first time in 1897 (2,8). Some other articles presented intranasal tooth as a nidus for developing rhinoliths (4-6). Almost all the cases in articles were unilateral and just one ectopic tooth, detected in each patient8 as in our case. Presentation of a tooth in nasal septum is extremely rare; as we know just one case has been reported by el-Sayed Y (9). 

 If abnormal tissue interactions disrupt the oral epithelium and the underlying mesenchymal tissue interactions process, the result is ectopic tooth development and eruption (10). It may be occur in mandibular condyle, coronoid process, orbit, palate, nasal cavity, nasal septum, chin and the maxillary antrum (11). Dentigerous cyst, crowded dentin and trauma and iatrogenic trauma may be other causes of it.

This condition is accompanied by variety of non specific symptoms which can postpone the diagnosis and therefore complications such as rhinosinusitis, osteomyelitis, dacryocystitis, nasal septal abscess, septal perforation, oronasal fistula and nasal deformity may develop (8). 

In Albert Chen and colleagues study they explain the clinic, as a characteristic and determining factor for diagnosing; but in our case physical exam was normal, except

a spur like septal deviation. We did not see any white mass in the nasal cavity; it may because of the site of nasal tooth.

Radiologic examinations, especially CT scan, can reveal the intranasal tooth and even the depth of eruption site1 (7,8,12). But in our case, radiological features were not definitely diagnostic, might be only suggestive in suspicious mind, because the tooth was too close to bone and discrimination of bone and tooth was very difficult.

However other articles suggested early removal of intranasal teeth (7,8), it seems that, because of the complications of septoplasty before completion of growth, like septal perforation and facial growth retardation, early surgical removal is not necessary in uncomplicated cases.

Our case was a patient with nasal obstruction and headache and positive history of sinusitis with septal deviation as main symptom. Clinical and radiological presentations were not characteristic; so we did not diagnose it until performing the surgery. After surgery all of the symptoms relieved. In this case surgery was both diagnostic and curative. 

In conclusion,ectopic tooth should be indiferential diagnosis of any radioopaque mass in imaging,such as osteoma and rhinolitis,great suspicion is essential for physician to consider this matter in patient with recurrent sinusitis or nasal obstruction 
